# Supplementary material for: Onopordum acanthium L. extract attenuates pancreatic β-Cells and cardiac inflammation in streptozocin-induced diabetic rats
Source: PLoS One. 2023 Jan 25;18(1):e0280464. doi: 10.1371/journal.pone.0280464 (PMC9876371; doi:10.1371/journal.pone.0280464)
Supplement: Attachment — Submitted filename: Response to Reviewers.docx [file pone.0280464.s002.docx]

**Dear Editor,**

**Thanks for this opportunity to publish our scientific research work in PLOS ONE , and thanks for your and the reviewers' valuable comments.**

**Responses to the reviewers' comments:**

**Reviewer #1**

-Check the abbreviations throughout the manuscript and introduce the abbreviation when the full word appears the first time in the text as well as abstract and then use only the abbreviation (For example, Streptozotocin (STZ), etc.,). Make a word abbreviated in the article that is repeated at least three times in the text, not all words need to be abbreviated.

**Response:**

**Ok, have been done with thanks**.

…………………………………………………………………………………….

-The full form of the species should be given when the first time appears in both the abstract and in the remaining part of the manuscript and it should be followed by only the first letter of the genus (e.g., Onopordum acanthium when the first time appears and followed by O. acanthium).

**Response:**

**Thanks, all notes have been followed and corrected.**

…………………………………………………………………………………….

The key words that are not in the title should also be included on the keywords. The keywords should assist computer searches to find your specific article.

**Response:**

**We have corrected and added with thanks.**

…………………………………………………………………………………….

-The technical terms (Latin Phrase) “in vivo”; “ex vivo” and “in silico” should be italic and it should be checked all over the manuscript.

**Response**

**We have corrected with thanks.**

…………………………………………………………………………………….

 -The authors may cite references for standard protocol, instead of mentioning kid or manufacture instructions, if a reference is given with it and the same should be added in the reference section.

**Response**

**All source references were added as links**

…………………………………………………………………………………….

-When referring to SPSS versions beginning from 19, authors should cite ‘IBM SPSS Statistics for Windows, version 22 (IBM Corp., Armonk, N.Y., USA)'.

**Response**

**-Kindly added.**

…………………………………………………………………………………….

-The table and figure legends should be improved and a proper footnote should be given. All legends should have enough description for a reader to understand the table and figure without having to refer back of the main text of the manuscript. For example, the necessary expansion may be given for abbreviations used. And also what does the superscripts (a,b,c,d,e, etc.) indicates.

**Response:**

**-We have followed and better explained with the indication of a,b,c, d, e… etc, as you mentioned with regards.**

…………………………………………………………………………………….

In the conclusion seems to be in general and is not given separately, it is highly recommended to include weaknesses of the study and potential future research goals in a separate heading.

**Response**

**We have re-written and corrected**

…………………………………………………………………………………….

**Reviewer #2**

-All comments raised are included in the manuscript. Authors should check for spell check and subject the manuscript to a native English speaker. Also, the resolution of all images is not clear enough.

**Response**

**Thank you, We have sent the manuscript to the proofreader in USA center and we have submitted a language proofreader certificate together with the manuscript. We have corrected all grammatical mistakes that you sent us.**

**Regarding the resolution, we reviewed and re-added the photos after rejection. This manuscript is a lot different from the previous one. We used pace in the PLOS ONE website to convert all images to the TIFF high quality images ( We have changed the image as a group by Pace website in TIF.**

…………………………………………………………………………………….

-How can streptozotocin be found in the methanolic extract from leaves of Onopordum acanthium (OAE). Justify?

**Response:**

**This section has been corrected and corrected all sentences.**

…………………………………………………………………………………….

-I did not see any ex vivo studies.

**Response:**

**Removed and you are right! thank you.**

…………………………………………………………………………………….

-Authors should justify why they selected OAE-200 and 400 mg/kg dosages

**Response:**

**Such doses have been applied before. We have checked as a start method and for our future work we will see higher and lower doses, we also worked a toxicity ,method.**

…………………………………………………………………………………….

-Why did the authors report in SE and not STD??

**Response**

**SEM is used instead of SD when the sample mean compared with the other means but SD only is used for description of data.**

-Catechin, Kaempferol and Epicatechin is the correct word

Response

All words have been corrected with thanks

…………………………………………………………………………………….

-What does a, b, c, d superscripts mean in the Tables 4, 5?

**Response**

**We have explained in this version below of each table 4 and 5.**

…………………………………………………………………………………….

**Highlights**

-All grammatical mistakes have been corrected

- All Corrected sentences and words have been highlighted in red color.

**Additional Editor Comments**

-It is preferable to include a positive control group.

**Response:**

**Mentioned and highlighted in red in this manuscript**

**Normal control as a negative control**

**Diabetic control as a positive control**

…………………………………………………………………………………….

-Please make sure that the words "in vivo" and "ex vivo" are italic throughout the manuscript.

**Response:**

**Checked and corrected.**

…………………………………………………………………………………….

-Please provide figure legends that have been misplaced.

**Response:**

**Corrected and explained.**

…………………………………………………………………………………….

-The individual figures need to be organized into groups

Response:

All figures have been organized into groups and saved as tif. Regards
