# Supplementary material for: Onopordum acanthium L. extract attenuates pancreatic β-Cells and cardiac inflammation in streptozocin-induced diabetic rats
Source: PLoS One. 2023 Jan 25;18(1):e0280464. doi: 10.1371/journal.pone.0280464 (PMC9876371; doi:10.1371/journal.pone.0280464)
Supplement: Attachment — Submitted filename: Reviewer.docx [file pone.0280464.s003.docx]

**Reviewer’s comments**

I have read the manuscript entitled “***Onopordum acanthium* extract attenuates pancreatic β-Cells and cardiac inflammation in streptozotocin-diabetic Rats**” several times and I have the following issues raised before the manuscript can be considered for possible publication

1. In the title the plant name is not the corrected way of writing. Authors should confirm and check the accepted using <http://mpns.kew.org/mpns-portal/?_ga=1.111763972.1427522246.1459077346> or <http://www.plantsoftheworldonline.org/> or <http://www.worldfloraonline.org/>.

- Also, in the title, streptozotocin-diabetic rats should be streptozotocin-induced diabetic rats

2. Abstract

-Did the author induced hypoglycemic associated inflammatory disorders

-the abbreviation of [OAE] does not represent methanolic extract of *Onopordum acanthium* (OAE).

- In the abstract line 28-30 should be rephrased and express correctly

-methods section not written properly. How many rats were used? How many groups did the authors used? Was there no standard drug group. If there was, how was the drug administered.

-line 36-38 not clear. Revise appropriately.

- Not enough biochemical assays was done to justify the study

-Under results insulin content, FBS and body weight alone is not enough to justify the study. More parameters need to be provided.

-line 42-43; STZ induced hyperglycemia associated inflammatory by the first sentence under background and objective shows it induces hypoglycemia associated inflammatory damage. Please clarify.

3. Introduction

-the accepted name of the plant should be written correctly.

- On the plant the anti-inflammatory activity has been reported before. What makes your work different from others.

-line 118 please revise

-line 122-123 should be moved above ……’To the best of our knowledge…..

Line 125-132 sentence does not make any sense. Authors should strive for clarity at all times. Revise

4. Methods

- The dosages of OAE 200 and 400 was not justified as well as the glibenclamide dose.

-DPPH assay to determine the antioxidant activity is not sufficient. Assays such as FRAP, Fe chelation, TAC, NO and OH radical scavenging should be carried out.

-Fasting blood glucose and body weight not enough.

-More parameters like serum insulin, glycated hemoglobin, HOMA-IR and HOMA-β should be carried out.

-in silico studies, the grid box for the docking should be provided.

-how were the ligands and proteins prepared. This should be separated and stated out clearly.

-Instead of the molecular docking, why didn’t the authors analyze for the induced-fit docking. Was the docking to the active site of the proteins or the authors did blind docking.

5. Discussion

- More recent studies can be used to improve the discussion especially the in silico section

6. Conclusion

-The analysis done is not sufficient enough to justify the conclusion made as well as the objectives of the study.

6. Conflict of interest

- The statement ‘The authors received no funding for this work’ is not appropriate here.

General comments

Authors should subject the manuscript to a native English speaker as most of the sentences are difficult to comprehend and also disjointed.

Thank you.
