# Supplementary material for: Onopordum acanthium L. extract attenuates pancreatic β-Cells and cardiac inflammation in streptozocin-induced diabetic rats
Source: PLoS One. 2023 Jan 25;18(1):e0280464. doi: 10.1371/journal.pone.0280464 (PMC9876371; doi:10.1371/journal.pone.0280464)
Supplement: Attachment — Submitted filename: Reviewer Comments 17102022.docx [file pone.0280464.s004.docx]

**Reviewer 3**

Thank you for inviting me to review this manuscript. I have the following comments:
1. Please, concise the abstract. It is too long.
2. The sentence lines 36-38 is an incomplete. Please, revise it.
3. Line 86: please, write β-cells in all manuscript sections. Not B-cells.
4. Line 193: Please, add ‘’Ethical approval’’ subtitle.
5. Line 215: replace the word ‘’divided’’ with the word ‘’allocated’’.
6. Line 232: in ‘’Experimental design’’, explain that 32 diabetic rats allocated into four groups other than the control non-diabetic (the fifth group).
7. Line 249: Please, replace the word ‘’measurement’’ with ‘’determination’’.
8. In Table 1, the authors used MRC1 Antibody as a human peptide, while your experiment was done on rats. Please, explain?????
9. In molecular docking, please, make it with protein of enzymes only, where you will determine if the ligands act as inhibitors or not.
10. Another comment regarding the molecular docking, the author made docking with protein of other species other than rat. Please, repeat it with rat proteins because your experiment was done on rats. But if you did not find the protein of rats, you can use the protein of human and make sequence alignment in https://www.uniprot.org/ to determine the degree of similarity between rat and human sequences of the same protein.
11. Please, write the pKi in Table 6 and 7.

**Reviewer 1**

The work entitled “Onopordum acanthium extract attenuates pancreatic β-Cells and cardiac inflammation in streptozotocin-diabetic Rats” is well designed and it contains different techniques including histology and immunohistochemistry, HPLC analyses, antioxidant activity and Molecular docking for testing of the anti-diabetic and cardio protective impact of extract from leaves of the O. acanthium. According to the authors’ results, the extract ameliorates negative effects of STZ. Finally, Authors discussed and proposed that rich bioactive compounds in the extract might have protective roles against to diabetic complications sourced from STZ. In my opinion, the manuscript can be accepted for publication but some minor remarks should be considered by authors before publication.

Minor comments:
Line 18-19: Delete the sententence "Streptozotocin (STZ) induces hypoglycaemia associated with inflammatory disorders." in Background and objective
Line 72: change the word “Diabetic” as “Diabetes”
Line 110: correct “( O.acanthium)” as “(O. acanthium)”
Line 136-137: Please indicate the name of the plant taxonomist who identified O. acanthium with a sentence.
Line 183: correct “per cent” as “percent”
Line 205: correct “Rattus norvegicus Domestica” as “Rattus norvegicus domestica” and as being italic.
Line 268: correct “deparaffinisation” as “deparaffinization”
Line 649: correct “Gallic“ as “gallic”
Line 687: correct “MiR-126” as “miR-126”

**Reviewer 2**

Reviewer’s comments
I have read the manuscript entitled “Onopordum acanthium extract attenuates pancreatic β-Cells and cardiac inflammation in streptozotocin-diabetic Rats” several times and I have the following issues raised before the manuscript can be considered for possible publication
1. In the title the plant name is not the corrected way of writing. Authors should confirm and check the accepted using http://mpns.kew.org/mpns-portal/?_ga=1.111763972.1427522246.1459077346 or http://www.plantsoftheworldonline.org/ or http://www.worldfloraonline.org/.
- Also, in the title, streptozotocin-diabetic rats should be streptozotocin-induced diabetic rats
2. Abstract
-Did the author induced hypoglycemic associated inflammatory disorders
-the abbreviation of [OAE] does not represent methanolic extract of Onopordum acanthium (OAE).
- In the abstract line 28-30 should be rephrased and express correctly
-methods section not written properly. How many rats were used? How many groups did the authors used? Was there no standard drug group. If there was, how was the drug administered.
-line 36-38 not clear. Revise appropriately.
- Not enough biochemical assays was done to justify the study
-Under results insulin content, FBS and body weight alone is not enough to justify the study. More parameters need to be provided.
-line 42-43; STZ induced hyperglycemia associated inflammatory by the first sentence under background and objective shows it induces hypoglycemia associated inflammatory damage. Please clarify.
3. Introduction
-the accepted name of the plant should be written correctly.
- On the plant the anti-inflammatory activity has been reported before. What makes your work different from others.
-line 118 please revise
-line 122-123 should be moved above ……’To the best of our knowledge…..
Line 125-132 sentence does not make any sense. Authors should strive for clarity at all times. Revise
4. Methods
- The dosages of OAE 200 and 400 was not justified as well as the glibenclamide dose.
-DPPH assay to determine the antioxidant activity is not sufficient. Assays such as FRAP, Fe chelation, TAC, NO and OH radical scavenging should be carried out.
-Fasting blood glucose and body weight not enough.
-More parameters like serum insulin, glycated hemoglobin, HOMA-IR and HOMA-β should be carried out.
-in silico studies, the grid box for the docking should be provided.
-how were the ligands and proteins prepared. This should be separated and stated out clearly.
-Instead of the molecular docking, why didn’t the authors analyze for the induced-fit docking. Was the docking to the active site of the proteins or the authors did blind docking.
5. Discussion
- More recent studies can be used to improve the discussion especially the in silico section
6. Conclusion
-The analysis done is not sufficient enough to justify the conclusion made as well as the objectives of the study.
6. Conflict of interest
- The statement ‘The authors received no funding for this work’ is not appropriate here.

General comments
Authors should subject the manuscript to a native English speaker as most of the sentences are difficult to comprehend and also disjointed.

Thank you.
